# Hearing impairment and frailty: The hidden psycho-cognitive-social mediating pathway

**DOI:** 10.1093/geroni/igaf122.1622

**Published:** 2025-12-31

**Authors:** Doris S F Yu, Chen Qiu

**Affiliations:** The University of Hong Kong, Hong Kong, China (People’s Republic); The University of Hong Kong, Hong Kong, China (People’s Republic)

## Abstract

Hearing impairment is highly prevalent problem affecting more than two-third of older adults worldwide. With more evidence to indicate its relationship with frailty, it is imperative to understanding the underlying mediating pathway to inform comprehensive care planning. Based on the biopsychosocial model, the objectives of this study were to: i) identify the relationship between hearing impairment and frailty, and ii) examine the mediating roles of depression, cognitive impairment and social loneliness in the relationship, if any From Dec 2023 to July 2024, a total of 2332 older adults were recruited from 7 geographic districts in Hong Kong. The Hearing Handicap Inventory for the Elderly-Screening version (HHIE-S) was used to assess the hearing impairment. The Edmonton Frail Scale (EFS) measured frailty from a multi-dimensional perspective. The 5-minute Montreal Cognitive Assessment Test (MoCA-5), Geriatric Depression Scale (GDS-15) and UCLA Loneliness Scale (UCLA3) were administered to measure the potential mediators. Structural equation model was conducted to test the hypothesized mediating model. The mean age of the participants was72.2 (SD = 7.1). The prevalence of hearing impairment and frailty were 13% and 16.4% respectively. The SEM result indicated that hearing impairment was directly associated with severe frailty [β = 0.147,95%CI(0.106-0.186)], and its relationship is also mediated through poor cognitive function [β = 0.011,95%CI(0.003-0.021)], social loneliness [β = 0.045,95%CI(0.031-0.061)] and depression [β = 0.034,95%CI(0.023-0.048)]. The indirect effect explained 38% of the total effect. This study recommended a holistic approach to manage late-life hearing impairment, with emphasis on compensatory communication strategies, brain health and social engagement optimization, and mental health support.

